# Fate by RNA methylation: m^6^A steers stem cell pluripotency

**DOI:** 10.1186/s13059-015-0609-1

**Published:** 2015-02-22

**Authors:** Boxuan Simen Zhao, Chuan He

**Affiliations:** Department of Chemistry and Institute for Biophysical Dynamics, The University of Chicago, 929 East 57th Street, Chicago, IL 60637 USA; Howard Hughes Medical Institute, The University of Chicago, 929 East 57 Street, Chicago, IL 60637 USA

## Abstract

The *N*^6^-methyladenosine (m^6^A) modification of mRNA has a crucial function in regulating pluripotency in murine stem cells: it facilitates resolution of naïve pluripotency towards differentiation.

## An old modification rediscovered: m^6^A in messenger RNA

**Figure 1:**
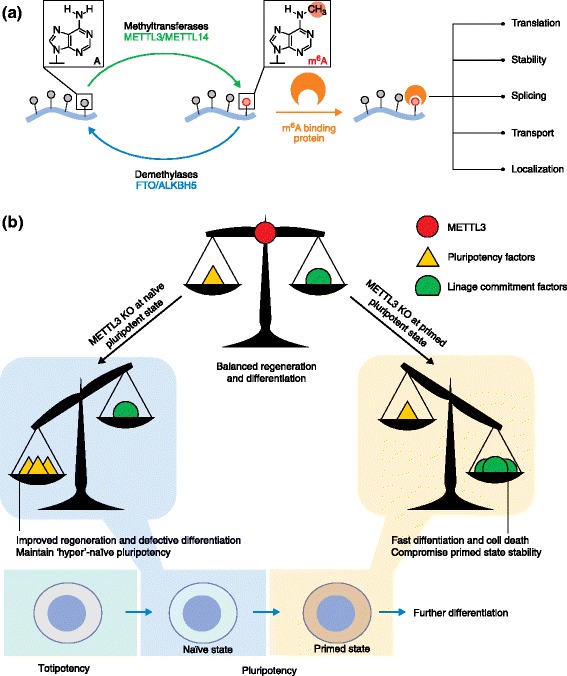
**The establishment and role of m** ^**6**^ **A RNA methylation in development. (a)** RNA methylation is dynamically regulated and impacts various aspects of RNA metabolism. The m^6^A marker is created by the activity of the m^6^A methyltransferase complex (METTL3-METTL14), and the methyl group can be reversibly removed by m^6^A demethylases; the modification can be recognized by m^6^A-binding proteins to effect biological functions. **(b)** Divergent effects of depletion through knockout (KO) of the N6-adenosine-methyltransferase subunit METTL3 during naïve and primed pluripotent states. As m^6^A reduces the stability of methylated transcripts, depletion of METTL3 in naïve pluripotent cells further upregulates the already-high naïve pluripotency genes to create a ‘hyper’-naïve pluripotent state, whereas depletion during the primed state further boosts the dominating lineage-commitment factors and tips the balance towards differentiation.

Epigenetic modifications of DNA and histones have been well investigated as crucial factors in the regulation of gene expression. However, the study of similar chemical modifications of RNA is still in its infancy. Among them, *N*^6^-methyladenosine (m^6^A) is the most abundant internal modification of the messenger RNA of almost all eukaryotes and of viruses that replicate in nuclei. Despite its existence being reported 40 years ago [1], the biological function and significance of m^6^A have only recently entered the research spotlight. The m^6^A modification was found to be dynamic, with the discovery of the alpha-ketoglutarate-dependent dioxygenase FTO and RNA demethylase ALKBH5, which are two demethylases that remove the methyl group from m^6^A within RNA, as well as the characterization of N^6^-adenosine-methyltransferase subunits METTL3 and METTL14, two methyltransferases that methylate adenosine residues to form m^6^A in RNA molecules [2-4] (Figure 1a). From these reports, methylation giving rise to m^6^A was demonstrated as the first confirmed reversible RNA modification, which sparked the re-emergence of research interest in this modification. Transcriptome-wide profiling of m^6^A further revealed its distribution pattern in mammalian cells and tissues [5,6]. It has a predominant sequence consensus and is strongly enriched around stop codons, and within long internal exons and at transcription start sites. These features suggest that m^6^A performs functional roles in regulation.

As the most abundant internal modification in mammalian mRNA, m^6^A is involved in multiple aspects of RNA metabolism, including RNA stability, translation, splicing, transport and localization (Figure 1a). One of the best-defined functions of m^6^A came alongside the discovery and characterization of the first m^6^A-specific binding protein, the YTH domain-containing family protein 2 (YTHDF2) [5,7]. YTHDF2 alters the overall stability of m^6^A-containing mRNAs by causing their relocalization to specialized mRNA-decay machineries, where they are committed to degradation. Other data also suggest roles for m^6^A in mRNA export and translation.

The modification m^6^A is essential in mammals and has been suggested to impact cell differentiation and development. Researchers have been probing the involvement of m^6^A in mouse and human embryonic stem cells (ESCs), but have produced somewhat conflicting results. Wang and colleagues [8] had reported in 2014 that knockdown of *Mettl3* and *Mettl14* in murine ESCs (mESCs) resulted in reduced m^6^A abundance and defective cell regeneration. Yet, in a more recent study, Batista and colleagues [9] found that complete knockout of *Mettl3* in mESCs led to improved self-renewal and resulted in blocked differentiation. In the latest paper on this topic, published in *Science*, Geula and colleagues [10] confirm the crucial roles of m^6^A methylation in the differentiation of mESCs and present a detailed demonstration of how depletion of m^6^A drives mESCs at naїve or primed pluripotency states to divergent fates.

## m^6^A as a fate determiner in murine embryonic stem cells

Geula and colleagues started by screening for factors essential for maintenance of pluripotency and identified several candidates. Among them, METLL3 emerged as a crucial component for regulation of stem cell pluripotency. The pluripotent stage can be further divided into two states: a ground, naїve state, and a differentiation-prepared, primed state. The authors then separately investigated the pluripotent stem cells at naїve (ESC) and primed (epiblast stem cell, EpiSC) states along with the respective impacts of m^6^A. To dissect the roles of METTL3 and m^6^A in the naїve pluripotent state, heterozygous *Mettl3*^*+/−*^ mice were generated, and homozygous *Mettl3*^*−/−*^ mESCs were obtained from embryos. In contrast to wild-type cells, *Mettl3*^*−/−*^ cells generated partially differentiated embryoid bodies (EBs), failed to produce mature neurons and were unable to proceed into a primed state upon corresponding inductions *in vitro*. Similarly, when these cells were injected into immunodeficient mice *in vivo*, the teratomas generated were poorly differentiated. These data indicate that the depletion of METTL3, and hence m^6^A, blocks differentiation in mESCs and keeps them in a so-called hyper-naїve state. By contrast, the depletion of METTL3 and m^6^A in the primed EpiSCs during a primed pluripotent state produced the opposite spectrum of effects - this depletion led to minimal self-renewal and fast differentiation, ultimately disrupting the stability of the primed state and resulting in cell death. Thus, the roles of depletion of METTL3 in this state are to reduce stem cell self-renewal and push them towards differentiation (Figure 1b).

This seemingly contradictory effect was explained by subsequent high-throughput sequencing analysis. Transcriptome-wide profiling of m^6^A (m^6^A-seq) was performed in ESCs, EBs and mature mouse cells. The results revealed that 80% of transcripts of naїve pluripotency genes and multiple lineage-commitment genes are methylated. Thus, m^6^A affects the genes governing both the naїve and primed states. Subsequent measurements of the global level and life-time of transcripts revealed that methylation shortens the half-life of modified mRNAs and reduces their abundance. Therefore, depletion of METTL3 increases the abundance and dominance of already-expressed genes, which leads to the observed phenotypes. For instance, in the naїve state, pluripotency genes predominate. Without m^6^A methylation, cells become stuck at the so-called hyper-pluripotent state, with high levels of expression of pluripotent transcripts. By contrast, at the primed state, lineage-commitment genes prevail, and removing m^6^A further tips the balance towards differentiation (Figure 1b).

Taking their findings further, the authors carefully measured the expression of both pluripotency and differentiation genes in *Mettl3*^−/−^ embryos before they died. It was found that the widespread expression of the pluripotency marker *Nanog* (encoding homeobox protein Nanog) was prolonged; and, although the expression of the differentiation marker *Pou5f1* (encoding transcription factor Oct4) was maintained, other lineage-commitment genes were not upregulated in the way that they are in wild-type embryos. Thus, knockout cells were in a naїve-like state, with a certain level of priming, exhibiting resistance to progression of differentiation. These *in vivo* findings are in good accord with the observed mESC phenotypes.

## Unifying mechanisms under a plethora of phenotypes

All the studies performed to date demonstrate that the presence of m^6^A reduces the stability of methylated mRNA transcripts in mESCs. Phenotypes are determined by the dominating type of transcripts, and m^6^A depletion works to intensify the trend. Although the authors did not explain how m^6^A affects mRNA stability, previous studies indicate that YTHDF2 could be partially responsible [6]. However, regulating mRNA stability is but one confirmed function of m^6^A. In this work, Geula and colleagues additionally suggest that m^6^A might increase the splicing efficiency of unfavored splicing events. Other aspects of RNA processing could also be affected by methylation and contribute to the observed phenotypes. In principle, with each discovered m^6^A-specific binding protein (or m^6^A 'reader'), there will be a corresponding function associated with m^6^A. Therefore, further research to identify and characterize m^6^A-specific binding proteins is important for uncovering the functions of mRNA m^6^A methylation. Studies of these reader proteins and their roles could provide underlying mechanisms for cell differentiation and development phenotypes associated with m^6^A.

## Concluding remarks

In summary, the comprehensive work presented by Geula *et al.* reveals m^6^A as a timely maintainer of the balance between pluripotency and lineage priming factors, thus ensuring orderly differentiation of mESCs. The authors have shown that m^6^A in mRNA might work as a ‘plug-in’ to other pre-existing pathways by altering downsteam gene expression. In this manner, RNA modifications can promote a fast response to external cues during times of cellular transformation or differentiation. We fully anticipate additional future discoveries that connect modifications of mRNA with the regulation of gene expression in cell differentiation and development in this fast-growing field.

## Abbreviations

EB: Embryoid body
EpiSC: Epiblast stem cell
mESC: Murine embryonic stem cell
m^6^A: *N*^6^-methyladenosine
